# The effects of dietary and lifestyle interventions among pregnant women with overweight or obesity on early childhood outcomes: an individual participant data meta-analysis from randomised trials

**DOI:** 10.1186/s12916-021-01995-6

**Published:** 2021-06-02

**Authors:** Jennie Louise, Amanda J. Poprzeczny, Andrea R. Deussen, Christina Vinter, Mette Tanvig, Dorte Moller Jensen, Annick Bogaerts, Roland Devlieger, Fionnuala M. McAuliffe, Kristina M. Renault, Emma Carlsen, Nina Geiker, Lucilla Poston, Annette Briley, Shakila Thangaratinam, Jodie M. Dodd

**Affiliations:** 1 The Robinson Research Institute, Discipline of Obstetrics and Gynaecology, The University of Adelaide, Adelaide, South Australia Australia; 2 Women’s and Babies Division, Department of Perinatal Medicine, The Women’s and Children’s Hospital, 72 King William Road, Adelaide, South Australia 5006 Australia; 3 Institute of Clinical Research University of Southern Denmark, 5230 Odense M, Denmark; 4 Department of Gynecology and Obstetrics, Odense University Hospital, Odense, Denmark; 5 Steno Diabetes Center, Odense University Hospital, 5000 Odense C, Denmark; 6 Department of Development and Regeneration, KU Leuven, Leuven, Belgium; 7 Faculty of Medicine and Health Sciences, Centre for Research and Innovation in Care (CRIC), University of Antwerp, Antwerp, Belgium; 8 Division of Mother and Child, Department of Obstetrics and Gynaecology, University Hospitals KU Leuven, Leuven, Belgium; 9 UCD Perinatal Research Centre, School of Medicine & Medical Science, University College Dublin, National Maternity Hospital, Dublin, Ireland; 10 Obstetric Clinic, Rigshospitalet, Copenhagen University Hospital, Copenhagen, Denmark; 11 Department of Obstetrics and Gynaecology, Hvidovre Hospital, University of Copenhagen, Hvidovre, Denmark; 12 Department of Pediatrics, Hvidovre University Hospital, Hvidovre, Denmark; 13 Department of Nutrition, Exercise and Sports, University of Copenhagen, Hvidovre, Denmark; 14 School of Life Course Sciences, Division of Women and Children’s Health, King’s College London, St. Thomas’ Hospital, London, UK; 15 Caring Futures Institute, College of Nursing and Health Sciences, Flinders University, Bedford Park, South Australia Australia; 16 WHO Collaborating Centre for Global Women’s Health, Institute of Metabolism and Systems Research, University of Birmingham, Birmingham, UK

**Keywords:** Individual participant data meta-analysis, Child follow-up of pregnancy intervention studies, Childhood obesity

## Abstract

**Background:**

The impact of maternal obesity extends beyond birth, being independently associated with an increased risk of child obesity. Current evidence demonstrates that women provided with a dietary intervention during pregnancy improve their dietary quality and have a modest reduction in gestational weight gain. However, the effect of this on longer-term childhood obesity-related outcomes is unknown.

**Methods:**

We conducted an individual participant data meta-analysis from RCTs in which women with a singleton, live gestation between 10^+0^ and 20^+0^ weeks and body mass index (BMI) ≥ 25 kg/m^2^ in early pregnancy were randomised to a diet and/or lifestyle intervention or continued standard antenatal care and in which longer-term maternal and child follow-up at 3–5 years of age had been undertaken. The primary childhood outcome was BMI *z*-score above the 90th percentile. Secondary childhood outcomes included skinfold thickness measurements and body circumferences, fat-free mass, dietary and physical activity patterns, blood pressure, and neurodevelopment.

**Results:**

Seven primary trials where follow-up of participants occurred were identified by a systematic literature search within the International Weight Management in Pregnancy (i-WIP) Collaborative Group collaboration, with six providing individual participant data. No additional studies were identified after a systematic literature search. A total of 2529 children and 2383 women contributed data. Approximately 30% of all child participants had a BMI *z*-score above the 90th percentile, with no significant difference between the intervention and control groups (aRR 0.97; 95% CI 0.87, 1.08; *p*=0.610). There were no statistically significant differences identified for any of the secondary outcome measures.

**Conclusions:**

In overweight and obese pregnant women, we found no evidence that maternal dietary and/or lifestyle intervention during pregnancy modifies the risk of early childhood obesity. Future research may need to target the pre-conception period in women and early childhood interventions.

**Trial registration:**

PROSPERO, CRD42016047165

**Supplementary Information:**

The online version contains supplementary material available at 10.1186/s12916-021-01995-6.

## Background

The World Health Organization has described childhood obesity as a serious public health challenge emerging in the twenty-first century [1]. With obesity occurring at increasingly earlier ages, so too does the aggregate lifetime exposure and risk of adverse health consequences [1]. Various inter-related factors, including parental genotype and shared obesogenic environment, contribute to an individual’s risk of obesity in childhood, and prenatal exposures, particularly high maternal body mass index (BMI), are important [2]. While the impact of maternal obesity extends beyond birth, being independently associated with an increased risk of early infant and childhood obesity [3], the extent to which the effect of maternal BMI may be modified by maternal diet and/or lifestyle remains unclear [2].

Global research efforts to date have focused on antenatal dietary and/or lifestyle interventions with the intention of limiting gestational weight gain (GWG) to improve health outcomes both for the woman and her infant. Our previous systematic review and individual participant data meta-analysis (IPDMA) of dietary and/or lifestyle interventions in pregnancy identified 36 RCTs, involving 15,526 women with a BMI of 18.5 kg/m^2^ and above [4]. The findings demonstrated that although women provided with a dietary intervention improved their dietary quality, there was evidence of only a modest reduction in GWG (0.7 kg). There was little evidence of an effect on the pre-specified composite maternal and infant outcomes, including birth weight [4]. Longer-term childhood outcomes such as BMI and obesity were not included [4].

From a Developmental Origins of Health and Disease (DOHaD) perspective, it is plausible that maternal dietary modification in pregnancy may have effects on the offspring which do not become evident until childhood [5]. To address this question, we performed an individual patient data meta-analysis (IPDMA) of randomised controlled trials (RCTs) in which women with overweight or obesity were provided with a dietary and lifestyle intervention during pregnancy and where follow-up of children had occurred to determine the longer-term effects of antenatal dietary and lifestyle intervention during pregnancy on the woman and their children at 3–5 years of age.

## Methods

### Study design

This IPDMA complied with the PRISMA-IPD guidelines and statement (Additional file 1) [6] and was prospectively registered with PROSPERO (ID number CRD42016047165) [7].

### Inclusion criteria for the studies and search strategy

Details have been published previously in our protocol [7]. In brief, individual patient data from RCTs in which women with a singleton, live gestation between 10^+0^ and 20^+0^ weeks, and of BMI ≥25 kg/m^2^ at the time of the first antenatal visit were randomised to receive a diet and/or lifestyle intervention or continued standard antenatal care and in which longer-term maternal and child follow-up at 3–5 years of age had been undertaken were eligible for inclusion. The included studies were identified by a systematic literature search within the International Weight Management in Pregnancy (i-WIP) Collaborative Group collaboration [4] and last updated March 2019 to ensure no additional studies had been overlooked. In addition, we searched PubMed (MEDLINE including ahead of print citations), PubMed Central (including bio-medical and life sciences journals and manuscripts submitted to comply with NIH open access policy), and Embase (includes MEDLINE and additional sources), through Ovid Medline, using search terms for dietary and lifestyle interventions in pregnancy, overweight, obesity, child cohort studies and childhood obesity (Additional File 2). The last search prior to data acquisition and analysis was undertaken in September 2019 and updated until March 2021.

### Data collection and management

As outlined in our protocol [7], each trial contributed de-identified participant-level data for each participant randomised, stored in a secure database. Variables included baseline descriptive information, allocated treatment intervention, and maternal pregnancy and birth outcomes, and neonatal outcomes were based on the original i-WIP individual participant data (IPD) [4].

Individual trial data, including missing data and randomisation processes, were checked to ensure consistency internally and with published reports. Initially, data from each trial were analysed separately and verified by the individual investigator before being incorporated into the combined database.

### Childhood variables collected through the i-WIP-3 Collaboration

An expanded database was created to include child height, weight, BMI, skinfold thickness measurements, calculated percentage body fat and fat-free mass, dietary and physical activity patterns, blood pressure, neurodevelopmental outcome domains and general health.

### Primary outcome measures

The primary childhood outcome was a BMI *z*-score above the 90th percentile, calculated using the WHO Child Growth Standards [8], determined by our Delphi survey [7]. We utilised a standardised online two-stage Delphi survey (February to April 2016) [9], to prioritise clinically relevant childhood outcomes. The panel involved members of the International Weight Management in Pregnancy Collaborative Group (iWIP) collaborative steering committee, members from the planned IPD investigators and other identified multidisciplinary experts in the field. The members scored each outcome using a Likert scale with a score of 9 considered critical, while a score of 1 was considered of limited importance to patient care. Members could suggest other outcomes which were included in the second round along with the highest scoring outcomes from the first round [7].

### Secondary outcome measures

A range of secondary maternal and childhood outcomes were assessed 3––5 years following birth, as described in our published protocol [5]. Secondary childhood outcome measures included height, weight, BMI, body circumferences (head, abdominal, and mid-upper arm), skinfold thickness measurements (SFTM) (subscapular, triceps, and biceps), fat mass and blood pressure. A parent-completed questionnaire was used to assess child dietary intake, physical activity, screen time and sleep time, with the parent-completed Ages and Stages Questionnaire used to screen child neurodevelopment [10]. Maternal secondary outcomes included weight, waist circumference, BMI, blood pressure and dietary intake assessed by a self-completed questionnaire. A number of additional measures were outlined in the protocol [7] but could not be reliably incorporated into the planned meta-analysis due to the extent of missing data and variable outcome definitions across the individual follow-up studies.

### Data management and statistical analyses

The primary analysis was based on the raw unimputed data. Our original intention was to base conclusions on results from analyses on multiply imputed data. However, issues with the imputation models necessitated the use of the raw data. Firstly, there was a high proportion (50% or more) of missing data for all outcomes, including some which were systematically missing in individual studies. Secondly, because all 3–5-year missing outcome data tended to be missing together, there were few auxiliary variables available to allow meaningful imputation of these outcomes. Additionally, one-stage (random effects) analyses for imputed data exhibited questionable convergence for almost all outcomes and non-convergence for some imputations. The decision was therefore made to use the raw data analyses as the primary analyses, with the imputed analyses as sensitivity analyses.

Analyses for all outcomes were performed using a one-stage (random effects) approach [11]. Mixed-effects models were fitted with fixed study-specific intercepts to allow for different baseline levels of outcome between studies, and a random intervention effect to allow for heterogeneity of intervention effect between studies. Covariates in adjusted models were fitted as fixed effects to avoid over-parameterisation and because there was no reason to expect these effects to differ between studies. Binary outcomes were analysed using mixed-effects log Poisson regression with robust variance, as the originally planned log binomial models did not converge. The effect of the intervention was estimated as a relative risk (RR) of the outcome (intervention vs control) and 95% confidence interval (CI). Continuous outcomes were analysed using mixed-effects linear regression models. The effect of the intervention was estimated as a difference in means (intervention − control) and 95% CI. Following recently published recommendations [12], restricted maximum likelihood (REML) estimation was used for linear regression models, with degrees of freedom calculated according to the Satterthwaite approximation. Analyses were undertaken using Stata v16 (StataCorp, College Station, TX).

To evaluate the robustness of the results of these analyses, a wide range of sensitivity analyses were performed. Firstly, alternative one-stage models were fitted in which study-specific intercepts were specified as random rather than fixed effects. Secondly, multiple imputation of child and maternal anthropometric outcomes was undertaken using two methods currently available for IPD of this nature: the two-stage fully conditional specification method [13], using the mice [14] and micemd [15] packages, and the joint multivariate normal method [16], using the jomo package [17], in R version 3.5 (R Foundation for Statistical Computing). For each imputation method, 100 complete datasets were created. Multiple imputation was performed separately by treatment group, with the imputation models including baseline variables (maternal age, BMI and parity at trial entry), pregnancy and birth variables (total GWG, gestational diabetes mellitus (GDM), gestational age (GA) at delivery, birth weight, length and head circumference (HC)) and child sex and age at follow-up. One-stage analyses of imputed data were performed in R v3.5, using the lme4 package [18] to fit models and the mitml package [19] to extract estimates.

Thirdly, two-stage analyses were performed for both raw and imputed data, in which estimates were first obtained separately for each study then combined using standard random-effects meta-analysis [11]. Between-study heterogeneity was estimated using the DerSimonian-Laird estimator; REML methods were also explored but led to convergence issues for some outcomes. Finally, analyses were performed in which the second intervention groups from the TOP [20] and Bogaerts [21] studies were excluded. The Bogaerts study included a second group receiving brochures and was included in the control group in the main analysis. The TOP study included a second group receiving a physical activity-only intervention and was included in the intervention group in the main analysis.

### Subgroup analyses

Subgroup analyses were planned to investigate the possibility of the differential effect of the intervention by maternal early pregnancy BMI category (25.0–29.9 vs ≥30.0kg/m^2^), parity (0 vs 1+) and ethnicity (Caucasian vs non-Caucasian). It was not possible to perform subgroup analyses by maternal ethnicity, as there were too few participants classed as non-Caucasian in all studies. Additionally, the analysis for maternal early pregnancy BMI had to be modified, as four of the six included studies only recruited women with BMI ≥30.0kg/m^2^, making the planned analysis impractical. The analyses were therefore carried out using BMI as a continuous variable. For parity, one study (ROLO) [22] could not be included for the estimation of the interaction effect, as only women with parity 1 were eligible for this study; however, data from ROLO were used to estimate the effect of the intervention in women with parity 1+.

Subgroup analyses were performed using a 2-stage approach only, due to convergence and collinearity issues when one-step models were correctly specified to separate across-study from within-study interaction effects [23, 24]. Within each study, a regression model was fitted including an interaction term between the subgroup (maternal early pregnancy BMI or parity) and intervention. The interaction effect was estimated, as well as the estimated effect of intervention at each level of the subgroup: for parity, the effect of the intervention on nulliparous and multiparous women and, for maternal BMI, the effect of the intervention at the mean BMI of 33.75kg/m^2^ and for an increase of 5kg/m^2^.

### Sample size

Power calculations were undertaken for the expected sample size and demonstrated acceptable power and coverage even for high levels of between-study heterogeneity. Full details can be found in the published protocol [7].

### Ethical considerations

Each participant in the individual trials and follow-up studies comprising the i-WIP-3 collaboration provided written informed consent to participate, with the data being used for the purposes for which the individual studies had approval. De-identified data were made available by the lead investigators of each trial.

### Patient and public involvement

No research participants, patients or members of the public were involved in the conceptualisation of this research study or setting the research question or outcome measures. They were not involved in the planning or implementation of this work, nor were they asked for advice or interpretation of the results.

## Results

**Fig. 1:**
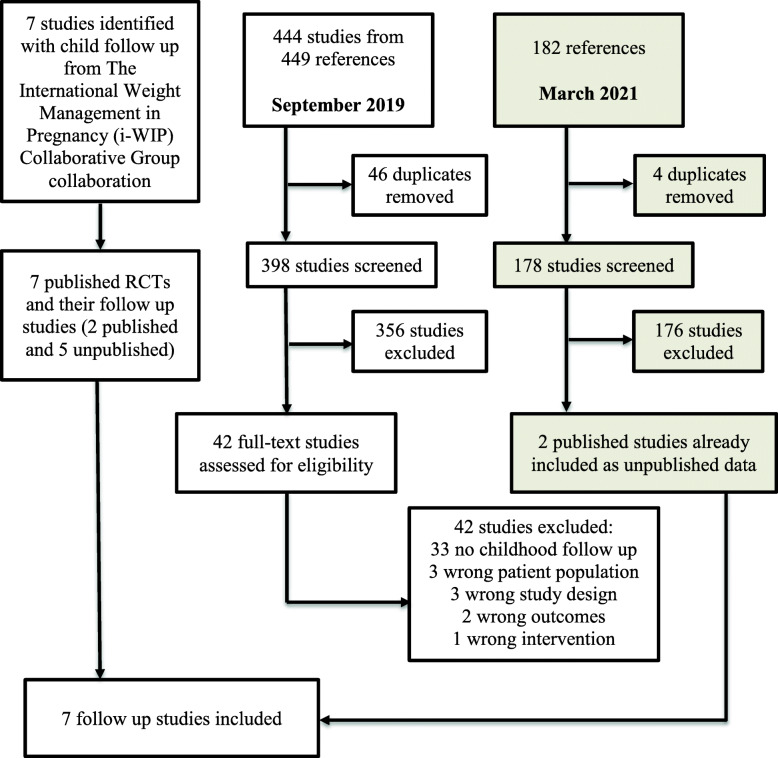
Flow chart of search results and study identification

A total of seven primary trials [20–22, 25–28] where follow-up of participants had occurred [29–32] were identified as eligible for inclusion in the IPDMA (Fig. 1), and the lead investigator approached to provide IPD. One study did not contribute IPD, due to lack of institutional permission to share data [28]. Of the 5180 women who participated in the original RCTs, 4800 women were considered eligible to participate in the 3–5-year follow-up studies (Table 1). Of these, 2529 children and 2383 women contributed at least one outcome variable (not necessarily the primary outcome). Maternal characteristics of participants contributing data were broadly similar between the intervention and control groups (Table 2) and similar to the baseline characteristics of all participants in the original RCTs (data not shown).

**Table 1:** Participant flow for the six studies included in the IPDMA

Characteristic	Total (all studies)	LIMIT	UPBEAT	ROLO	TOP^d^	LiP	Bogaerts^c^
Randomised to original RCT^a^							
- Control	2550	1104	772	226	141	180	127
- Intervention	2638	1108	783	205	284	180	78
- Overall	5180	2212	1555	431	425	360	205
Eligible for 3–5-year follow-up^b^							
- Control	2362	1056	751	226	133	75	121
- Intervention	2438	1065	759	204	253	81	76
- Overall	4800	2121	1510	430	386	156	197
Participated (children)^e^							
- Control	1237	691	263	107	44	75	57
- Intervention	1292	726	250	107	89	81	39
- Overall	2529	1417	513	214	133	156	96
Participated (mothers)^e^							
- Control	1159	626	260	107	42	67	57
- Intervention	1224	663	251	107	88	76	39
- Overall	2383	1289	511	214	130	143	96

^a^Includes only randomised participants with BMI ≥25.0 kg/m^2^

^b^Eligible for follow-up if there was a known live birth, no known infant or maternal death, and has not withdrawn from the study with the withdrawal of permission to use data

^c^Bogaerts study included 3 groups: control, diet and physical activity intervention and brochures. Women randomised to the brochures group have been included under Control for the purposes of the IPDMA

^d^TOP study included 3 groups: control, diet and physical activity intervention and physical activity alone. Women randomised to the physical activity only intervention have been included under Intervention for the purposes of the IPDMA

^e^‘Participated’ means that at least one outcome was available of those analysed as part of the 3–5-year follow-up (not necessarily primary outcome)

**Table 2:** Baseline characteristics of women at trial entry and children at follow-up

Characteristic	Overall, *n*=2535	Intervention, *n*=1297	Control, *n*=1238
BMI category, *N* (%)			
- 25.0–29.9	790 (31.16)	389 (29.99)	401 (32.39)
- 30.0–34.9	954 (37.63)	501 (38.63)	453 (36.59)
- 35.0–39.9	516 (20.36)	260 (20.05)	256 (20.68)
- ≥40.0	275 (10.85)	147 (11.33)	128 (10.34)
Maternal BMI (kg/m^2^), median (IQR)	32.32 (28.80, 35.90)	32.40 (29.00, 35.97)	32.29 (28.70, 35.80)
Maternal age (years), mean (SD)	30.46 (5.19)	30.39 (5.18)	30.52 (5.19)
Parity, *N* (%)			
- 0	1060 (41.81)	558 (43.02)	502 (40.55)
- 1+	1475 (58.19)	739 (56.98)	736 (59.45)
Maternal height (cm), mean (SD)	165.28 (6.62)	165.45 (6.61)	165.11 (6.64)
Maternal weight (kg), mean (SD)	90.39 (16.66)	90.88 (16.54)	89.88 (16.77)
Ethnicity, *N* (%)			
- Non-Caucasian	278 (11.07)	134 (10.43)	144 (11.75)
- Caucasian	2233 (88.93)	1151 (89.57)	1082 (88.25)
Infant sex, *N* (%)			
- Male	1263 (49.82)	650 (50.12)	613 (49.52)
- Female	1272 (50.18)	647 (49.88)	625 (50.48)
Child age (years) at follow-up, mean (SD)	3.56 (0.83)	3.53 (0.79)	3.59 (0.86)

### Child anthropometric outcomes

#### Primary outcome

Approximately 30% of all child participants had a BMI *z*-score above the 90th percentile, although there was no significant difference between the intervention and control groups (adjusted relative risk (aRR) 0.97; 95% CI 0.87, 1.08; *p*=0.610) (Table 3). There was no evidence of substantial heterogeneity of the intervention effect between studies (estimated *τ*^2^=0.00).

**Table 3:** Child anthropometric outcomes

Outcome	Intervention, *n*=1292^f^	Control, *n*=1237^f^	Unadjusted estimate (95% CI)	Unadjusted *p*	*τ* unadjusted model^d^	Adjusted estimate (95% CI)	Adjusted *p*	*τ* adjusted model^d^
BMIz >90th percentile^a^	380/1262 (30.11)	355/1208 (29.39)	0.84 (0.53, 1.33)	0.449	0.24	0.97 (0.87, 1.08)	0.610	0.00
Weight (kg)^b^	16.79 (2.94)	16.88 (3.30)	2.80 (−4.37, 9.97)	0.362	6.70	0.17 (−1.19, 1.52)	0.341	0.00
Height (cm)^b^	100.07 (6.81)	100.49 (7.29)	16.85 (−26.48, 60.19)	0.363	40.56	1.00 (−0.93, 2.94)	0.132	0.31
Head circumference (cm)^b^	50.46 (1.78)	50.48 (1.74)	12.78 (−27.42, 52.99)	0.386	25.02	0.54 (−1.04, 2.11)	0.204	0.25
BMI (kg/m^2^)^b^	16.66 (1.54)	16.58 (1.50)	2.94 (−4.38, 10.26)	0.349	6.85	0.24 (−0.15, 0.63)	0.136	0.15
Weight *z*-score^c^	0.69 (0.98)	0.64 (1.01)	0.19 (−0.13, 0.52)	0.186	0.27	0.05 (−0.53, 0.63)	0.446	0.00
Height *z*-score^c^	0.19 (0.99)	0.18 (1.01)	0.09 (−734.51, 734.69)	0.540	0.00	–	–	–^e^
Head circumference *z*-score^c^	0.76 (1.16)	0.74 (1.11)	0.20 (−0.43, 0.82)	0.370	0.32	0.04 (−0.45, 0.53)	0.701	0.11
BMI *z*-score^c^	0.84 (1.04)	0.78 (1.02)	0.22 (−0.21, 0.64)	0.243	0.37	0.05 (−0.55, 0.66)	0.427	0.00
Weight for length (WFL) *z*-score^c^	0.84 (1.04)	0.78 (1.03)	0.21 (−0.28, 0.69)	0.319	0.40	0.07 (−1.56, 1.70)	0.425	0.00
Abdomen circumference (cm)^b^	52.13 (4.04)	52.08 (4.30)	8.97 (−13.64, 31.57)	0.355	21.15	0.65 (−0.41, 1.72)	0.133	0.37
Arm circumference (cm)^b^	17.22 (1.61)	17.10 (1.60)	5.77 (−18.40, 29.93)	0.412	9.68	0.27 (−5.66, 6.19)	0.311	0.00*
Biceps skinfold (mm)^b^	7.23 (2.70)	7.16 (2.62)	1.72 (−4.16, 7.61)	0.420	3.64	0.10 (−2.55, 2.75)	0.572	0.00*
Triceps skinfold (mm)^b^	11.40 (3.30)	11.22 (3.13)	2.01 (−3.24, 7.26)	0.370	4.89	0.27 (−0.50, 1.04)	0.238	0.12
Subscapular skinfold (mm)^b^	6.85 (2.29)	6.87 (2.51)	1.12 (−1.90, 4.14)	0.384	2.81	0.02 (−0.45, 0.48)	0.910	0.17
Fat mass (kg)^b^	3.91 (1.41)	3.95 (1.36)	0.95 (−1.30, 3.19)	0.306	1.76	0.03 (−0.89, 0.95)	0.737	0.00
Systolic blood pressure (mmHg)^b^	100.05 (8.62)	100.18 (8.52)	17.48 (−26.55, 61.51)	0.354	41.15	1.33 (−2.15, 4.82)	0.180	0.00*
Diastolic blood pressure (mmHg)^b^	60.49 (8.60)	59.92 (8.44)	11.03 (−15.78, 37.84)	0.338	25.04	1.00 (−0.95, 2.95)	0.205	0.87

^a^Binary outcome: descriptives are number/total and percentage; estimates are relative risk of BMI *z*-score > 90th percentile (intervention/control) and 95% confidence interval from a mixed-effects GLM (log Poisson with robust variance)

^b^Continuous outcome: descriptives are mean (SD); estimates are difference in means (intervention − control) and 95% confidence interval from a linear mixed model. Adjusted models included maternal BMI, parity (0 vs 1+), age, child sex and actual age at follow-up as covariates

^c^Continuous outcome: descriptives and estimates as above. Adjusted models included maternal BMI, parity (0 vs 1+) and age at trial entry as covariates

^d^Estimated standard deviation of the random intervention effect (assumed ~ *N*(0, *τ*^2^)), indicating heterogeneity of treatment effect. Estimates marked with ‘*’ were extremely unstable with very large standard errors and should therefore be interpreted with caution

^e^Adjusted model did not converge

^f^Number of children from all studies for whom data on any 3–5-year follow-up outcome was available

#### Secondary outcomes

There were no statistically significant or clinically meaningful differences between the intervention and control groups in relation to any secondary child anthropometric outcomes. Estimates of the heterogeneity of the intervention effect were small but demonstrated instability, with very large standard errors for some outcomes.

#### Sensitivity analyses

Removing the second intervention groups from the Bogaerts [21] and TOP [20] studies had no effect on the results of the analyses. Likewise, the results were not changed when random intercepts were substituted for fixed intercepts in the one-step model, or when two-step analyses were used. In one-step analyses of imputed data, the results were overall unaffected, with the exception of head circumference (HC) measurement in the data imputed using the 2-stage fully conditional specification (FCS) method, where the intervention group had higher HC by 5.91 (95% CI 0.17, 11.66; cm, *p*=0.044). However, the estimated heterogeneity of the intervention effect was high (at 79.83), and this outcome was also one which was systematically missing (not collected) for two studies (LiP [27] and Bogaerts [21]), with the imputation model for this outcome considered unreliable.

### Maternal anthropometric outcomes

There were no statistically significant or clinically meaningful differences between the intervention and control groups in relation to any of the maternal anthropometric outcomes (Table 4). Estimates of between-study heterogeneity of intervention effect were small but demonstrated some instability.

**Table 4:** Maternal anthropometric outcomes

Outcome	Intervention (*n*=1224)^d^	Control (*n*=1159)^d^	Unadjusted estimate (95% CI)	Unadjusted *p*	*τ* unadjusted model^c^	Adjusted estimate (95% CI)	Adjusted *p*	*τ* adjusted model^c^
Weight (kg)^a^	92.63 (18.78)	91.62 (19.30)	16.13 (−22.71, 54.97)	0.335	36.37	0.70 (−2.95, 4.35)	0.389	0.00
Waist circumference (cm)^a^	99.49 (14.31)	99.47 (14.43)	23.86 (−53.94, 101.65)	0.401	48.43	0.82 (−3.36, 5.00)	0.338	0.00*
BMI (kg/m^2^)^a^	33.92 (6.36)	33.65 (6.60)	5.89 (−8.59, 20.38)	0.343	13.57	0.22 (−0.87, 1.32)	0.366	0.00
Systolic blood pressure (mmHg)^a^	119.83 (12.06)	120.75 (12.94)	28.91 (−68.27, 126.10)	0.413	60.35	0.55 (−6.95, 8.04)	0.599	0.00*
Diastolic blood pressure (mmHg)^a^	75.22 (9.40)	76.06 (9.95)	17.92 (−43.89, 79.73)	0.424	38.38	−0.13 (−4.51, 4.25)	0.872	0.64
Weight change^b^ trial entry until follow-up	1.23 (10.23)	0.86 (10.17)	0.86 (−2.51, 4.22)	0.268	0.43	0.52 (−2.47, 3.51)	0.424	0.00*

^a^Continuous outcome: descriptives are mean (SD); estimates are difference in means (intervention − control) and 95% confidence interval from a linear mixed model. Adjusted models included maternal BMI, parity (0 vs 1+), age at trial entry and elapsed time between delivery and follow-up

^b^Continuous outcome: descriptives and estimates as above. Adjusted models included maternal BMI, parity (0 vs 1+), weight and age at trial entry and elapsed time since delivery

^c^Estimated standard deviation of the random intervention effect (assumed ~ *N*(0, *τ*^2^)), indicating heterogeneity of treatment effect. Estimates marked with ‘*’ were extremely unstable with very large standard errors and should therefore be interpreted with caution

^d^Number of women from all studies for whom data on any 3–5-year follow-up outcome was available

These results were not altered for any of the sensitivity analyses. Specifically, there were no significant differences where the second intervention groups from TOP [20] and Bogaerts [21] studies were removed, where random study intercepts were substituted for fixed intercepts, in the 2-step analyses or in analyses of the imputed data (data not shown).

### Child diet, activity and development outcomes

There were no statistically significant or clinically meaningful differences between the intervention and control groups in relation to any of the child diet, activity or development outcomes (Table 5). Estimates of heterogeneity of intervention effect were small for most outcomes, but with some substantial heterogeneity observed for screen time, and for some Ages and Stages scores.

**Table 5:** Child diet, activity and developmental outcomes

Outcome	Intervention, *n*=1292^e^	Control, *n*=1237^e^	Unadjusted estimate (95% CI)	Unadjusted *p*	*τ* unadjusted model^d^	Adjusted estimate (95% CI)	Adjusted *p*	*τ* adjusted model^d^
Ever breastfed^a^	1049/1365 (76.85)	986/1300 (75.85)	0.93 (0.89, 0.98)	0.003	0.00	1.01 (0.98, 1.05)	0.495	0.00
Breastfed ≥6months^a^	477/1016 (46.95)	470/968 (48.55)	0.79 (0.58, 1.07)	0.126	0.07	0.97 (0.93, 1.01)	0.146	0.00
Energy (kJ)^b^	4427.56 (1404.86)	4358.04 (1354.44)	1635.52 (−4661.88, 7932.91)	0.380	2518.13	110.99 (−1908.35, 2130.32)	0.394	0.15*
Fat/day (g)^b^	36.30 (12.38)	35.41 (11.74)	13.71 (−35.27, 62.69)	0.352	19.57	1.82 (−4.39, 8.02)	0.312	1.93
Protein/day (g)^b^	43.41 (15.39)	42.24 (14.17)	16.17 (−43.25, 75.59)	0.362	23.75	1.53 (−19.70, 22.76)	0.330	0.00*
Carbohydrate/day (g)^b^	168.18 (71.61)	171.28 (73.59)	47.57 (−148.78, 243.92)	0.407	78.44	1.32 (−80.57, 83.21)	0.723	0.00*
Fruits/day^c^	3.28 (2.07, 4.79)	3.12 (2.00, 4.56)	1.28 (−2.32, 4.88)	0.340	2.22	0.22 (−1.60, 2.04)	0.318	0.00*
Vegetables/day^c^	2.79 (1.64, 4.64)	2.93 (1.72, 4.43)	1.10 (−2.21, 4.40)	0.369	2.04	0.17 (−1.53, 1.86)	0.370	0.00*
Dairy/day^c^	2.57 (1.71, 3.64)	2.43 (1.57, 3.50)	0.90 (−1.46, 3.25)	0.312	1.46	0.15 (−0.28, 0.59)	0.288	0.15
Extras/day^c^	1.64 (0.93, 2.57)	1.71 (0.93, 2.65)	0.45 (−0.95, 1.85)	0.381	0.85	−0.05 (−1.09, 1.00)	0.616	0.00*
Physical activity, (min/week)^c^	982.50 (570.00, 1650.00)	915.00 (510.00, 1680.00)	576.87 (−1795.95, 2949.70)	0.405	946.32	41.55 (−1157.17, 1240.27)	0.522	0.00*
Screen time (min/week)^c^	400.00 (210.00, 720.00)	390.00 (210.00, 630.00)	204.40 (−647.94, 1056.74)	0.410	339.19	20.71 (−173.60, 215.02)	0.515	23.55
Ages and stages communication^c^	55.00 (50.00, 60.00)	55.00 (50.00, 60.00)	13.92 (−29.36, 57.19)	0.381	26.45	0.62 (−4.6e+07, 4.6e+07)	0.764	0.00*
Ages and stages gross motor^c^	60.00 (50.00, 60.00)	60.00 (50.00, 60.00)	13.89 (−31.82, 59.60)	0.404	27.98	0.84 (−5.2e+07, 5.2e+07)	0.728	0.00*
Ages and stages fine motor^c^	50.00 (40.00, 60.00)	50.00 (40.00, 60.00)	9.53 (−32.40, 51.46)	0.521	25.65	−1.62 (−8.26, 5.03)	0.458	2.95
Ages and stages problem solving^c^	60.00 (50.00, 60.00)	60.00 (50.00, 60.00)	14.00 (−30.60, 58.59)	0.391	27.27	1.14 (−8.8e+07, 8.8e+07)	0.707	0.00*
Ages and stages personal social^c^	55.00 (50.00, 60.00)	55.00 (50.00, 60.00)	14.22 (−29.01, 57.46)	0.372	26.44	1.11 (−3.26, 5.48)	0.407	1.60
Ages and stages total^c^	270.00 (250.00, 285.00)	270.00 (245.00, 285.00)	64.76 (−154.29, 283.82)	0.416	134.10	1.32 (−21.00, 23.63)	0.786	5.46
Total sleep/night (h)^c^	11.16 (1.25)	11.16 (1.27)	3.77 (−12.38, 19.92)	0.421	6.47	0.18 (−2.31, 2.68)	0.323	0.00*

^a^Descriptives are number/total and percentage; estimates are relative risk (intervention/control) and 95% confidence interval from a mixed-effects GLM (log Poisson with robust variance)

^b^Descriptives are mean (SD); estimates are difference in means (intervention − control) and 95% confidence interval from a linear mixed model. Adjusted models included maternal BMI category, parity (0 vs 1+), age at trial entry, child sex and actual age at follow-up

^c^Descriptives are median (IQR); estimates are difference in means (intervention − control) and 95% confidence interval from a linear mixed model. Adjusted models included maternal BMI, parity (0 vs 1+), age at trial entry, child sex and actual age at follow-up

^d^Estimated standard deviation of the random intervention effect (assumed ~ *N*(0, *τ*^2^)), indicating heterogeneity of treatment effect. Estimates marked with ‘*’ were extremely unstable with very large standard errors and should therefore be interpreted with caution

^e^Number of children from all studies for whom data on any 3–5-year follow-up outcome was available

The sensitivity analyses undertaken for these outcomes comprised 2-step analyses, analyses substituting random intercepts for fixed study intercepts and analyses excluding the second intervention groups from TOP [20] and Bogaerts [21] (only for those outcomes which were collected in those studies). The results were not affected by any of these sensitivity analyses.

### Maternal diet outcomes

There was no evidence of an effect of intervention on any of the maternal diet outcomes (Table 6). In general, there was evidence of substantial between-study heterogeneity, reflecting the differences in the questionnaires used to collect these outcomes. Sensitivity analyses—2-stage analyses and analyses substituting random intercepts for fixed study intercepts—did not affect the results.

**Table 6:** Maternal diet outcomes

Outcome	Intervention (*n*=1224)^b^	Control (*n*=1159)^b^	Unadjusted estimate (95% CI)	Unadjusted *p*	*τ* unadjusted model^c^	Adjusted estimate (95% CI)	Adjusted *p*	*τ* adjusted model^c^
Mother energy (kJ)^a^	7649.98 (3232.58)	7705.48 (2832.20)	2490.10 (−9919.95, 14900.15)	0.479	4966.20	−111.83 (−1496.28, 1272.61)	0.736	404.07
Mother fat (g)^a^	65.87 (30.64)	66.96 (27.88)	20.77 (−87.10, 128.63)	0.494	43.15	−1.74 (−15.90, 12.42)	0.626	4.45
Mother protein (g)^a^	93.16 (42.61)	92.51 (35.92)	31.39 (−111.14, 173.91)	0.443	56.98	1.11 (−40.42, 42.64)	0.668	0.01*
Mother carbohydrate (g)^a^	221.42 (105.69)	223.80 (94.77)	70.96 (−293.58, 435.51)	0.490	145.81	−4.09 (−49.76, 41.59)	0.706	12.95
Mother fruits^a^	2.07 (1.14, 3.64)	2.00 (1.07, 3.29)	1.14 (−2.82, 5.10)	0.338	1.51	0.36 (−2.16, 2.88)	0.591	0.86
Mother vegetables^a^	4.93 (2.93, 7.71)	4.86 (3.00, 7.64)	2.02 (−7.29, 11.33)	0.449	3.68	0.18 (−2.81, 3.18)	0.804	0.96
Mother dairy^a^	1.50 (0.86, 2.57)	1.50 (0.93, 2.57)	0.68 (−2.41, 3.76)	0.444	1.22	0.02 (−1.93, 1.98)	0.841	0.00
Mother extras^a^	1.14 (0.64, 2.00)	1.21 (0.70, 2.21)	0.07 (−3.68, 3.82)	0.943	1.47	−0.41 (−2.92, 2.11)	0.555	0.94

^a^Descriptives are mean (SD); estimates are difference in means (intervention − control) and 95% confidence interval from a linear mixed model. Adjusted models included maternal BMI category (25.0–29.9 vs ≥30.0), parity (0 vs 1+), age and elapsed time between delivery and follow-up

^b^Number of mothers from all studies for whom data on any 3–5-year follow-up outcome was available

^c^Estimated standard deviation of the random intervention effect (assumed ~ *N*(0, *τ*^2^)), indicating heterogeneity of treatment effect. Estimates marked with ‘*’ were extremely unstable with very large standard errors and should therefore be interpreted with caution

### BMI subgroup analyses

There was no evidence of a differential effect of intervention by maternal early pregnancy BMI for any of the child anthropometric outcomes. However, several of the interaction terms for maternal anthropometric outcomes at 3–5-year follow-up were statistically significant, including maternal BMI (interaction effect −0.07 (−0.14, −0.00), *p*=0.044), maternal waist circumference (interaction effect −0.20 (−0.40, −0.01), *p*=0.044), maternal diastolic blood pressure (interaction effect −0.20 (−0.39, −0.00), *p*=0.045) and maternal weight change from trial entry to follow-up (interaction effect −0.19 (−0.38, −0.01), *p*=0.038).

There is some evidence to suggest that as maternal BMI increases, maternal 3–5-year follow-up measures in the intervention group decrease relative to those in the control group. However, overall, the estimates of the difference between the intervention and control groups at the overall mean BMI (33.8kg/m^2^) were not statistically significant. These results should be interpreted with a high degree of caution as the *p* values have not been adjusted for multiple comparisons; this is a secondary, exploratory analysis and the effect size is modest.

In sensitivity analyses on imputed data, these interaction effects were observed in data imputed using the multivariate normal (MVN) method, but not in data imputed using the 2-stage FCS method.

### Parity subgroup analyses

There was no evidence of a differential effect of the intervention by parity on any child or maternal anthropometric outcome, in either the main analyses or the sensitivity analyses.

## Discussion

### Overall findings

Our findings demonstrate that pre-school aged children born to women with overweight or obesity during pregnancy are themselves at risk of high BMI, with approximately 30% of the cohort having a BMI *z*-score above the 90th percentile. While many of the individual trials identified pregnancy intervention to be associated with improvements in maternal diet [22, 25, 26, 33, 34], and a reduction in risk of high infant birth weight [25, 35], there was no evidence of an effect on childhood weight, adiposity, or dietary and physical activity patterns at 3–5 years of age. Furthermore, there was no evidence of a persistent difference in maternal weight 3–5 years after pregnancy, despite modest differences in GWG evident from some studies [21, 22, 25, 26, 33]. These findings are robust, with the original trials being conducted in different countries across the globe, and despite considerable variation in terms of the intensity of the intervention ranging from three [22] up to weekly sessions [27] across pregnancy.

### Strengths and limitations

By combining and analysing the extensive volume of RCT data available, we have been able to evaluate the longer-term maternal and childhood health outcomes with sufficient statistical power, while avoiding the expense, duplication of effort and inevitable time delays which would have occurred by undertaking another large-scale RCT with pre-specified primary outcomes relating to longer-term maternal or child health. The sample size of 2529 child participants represents the largest prospectively collected data set available from participants of randomised trials during pregnancy, with a standardised assessment of anthropometric measures, and consistent evaluation of dietary, physical activity, sedentary behaviour and sleep patterns, all of which are well-recognised early life factors contributing to child overweight and obesity [36].

Our study is not without limitations. Despite the agreement by investigators of pre-specified outcomes, and the a priori generation of our protocol [7], there were a number of measures that could not be reliably incorporated into the meta-analysis due to the extent of missing data and variable outcome definitions across the individual follow-up studies. Furthermore, there is a potential risk of selection bias. Of the total eligible randomised cohort, there was a considerable variation in the proportion of children assessed and who contributed data, ranging from approximately 34% [20, 26] up to 67% [25]. However, baseline and clinical characteristics of women and children for whom data were available and who participated in the follow-up studies were similar between the randomised intervention and control groups and also similar to the full randomised cohort. Sensitivity analyses were conducted under a wide range of different scenarios, with the findings consistent under a variety of plausible assumptions. On balance, therefore, we do not consider the risk of bias to be significant, and any potential impact on the validity of our findings is low.

A further potential limitation of our trial is the generalisability and external validity of our findings. Across all trials, the population was approximately 90% Caucasian, precluding our ability to evaluate the role of maternal ethnicity as we had originally proposed [7]. This continues to be a limitation, with the available randomised trial literature to date predominantly recruiting women who are Caucasian [4].

While appropriate statistical methodology to evaluate IPDMA continues to evolve, we utilised approaches as recommended in the most recent literature and conducted sensitivity analyses where there was any question about the most appropriate methods (for example, in the imputation of missing data). Nevertheless, we encountered numerous challenges in implementing these methods in practice, from imputation for a small number of trials where a very large proportion of data are missing and auxiliary data are not consistently available, to convergence issues with one-stage meta-analysis models.

### Findings into context with the literature

We are aware of a number of trials [22, 26, 27] that have conducted and reported findings of childhood follow-up at 6 months [37–39], 18 months [31, 40–43] and 3–5 years [30, 44] after birth. Together, findings from the individual studies alone and when incorporated into the IPDMA suggest little longer-term effect on child BMI and adiposity measures.

However, we have demonstrated that children born to women with overweight or obesity during pregnancy themselves remain at risk of early childhood overweight and obesity. This cohort of pre-school aged children has a prevalence of BMI *z*-score above the 90th percentile of approximately 30%. This is in contrast to data reported from the broader childhood population in Australia [45] and Europe [46] where a combined total of 20 [45] to 30% [46] of pre-school aged children are overweight or obese.

Our study also demonstrates the frequent occurrence of obesogenic behaviours, even at age 3–5 years, with the majority of children not meeting the recommended number of daily servings of vegetables, while exceeding both fruit intake and discretionary food intake [47]. Furthermore, the majority of children who contributed data to this IPD-MA did not meet physical activity recommendations of at least 3 h per day and were at the upper range of the 1 h screen time per day [48].

These findings are broadly consistent with dietary intake data from Australian children aged 4–8 years who have similarly poor consumption of vegetables in particular, while far exceeding intake of calorie-dense discretionary foods [49]. The health benefits of fruit and vegetable consumption, even from an early age, are well recognised [50], with their consumption from infancy [51] contributing to persistent sub-optimal eating habits in later childhood and adolescence [51].

Current clinical recommendations internationally advocate intervention in pregnancy [52] through improved diet and limiting gestational weight gain. While healthy diet and physical activity in pregnancy are prudent [53], a significant paradigm shift is required if maternal and child health is to be improved, particularly in relation to child obesity. A continued focus on intervening in pregnancy and a relentless search for the illusory effective pregnancy dietary and/or lifestyle intervention is unlikely to be successful in light of the amassed randomised trial evidence both during pregnancy [4] and now extending into childhood.

A timely opportunity exists in which to refocus research efforts towards ensuring optimal maternal health and weight prior to conceiving, as well as ongoing evaluation of the role of early childhood interventions. This will undoubtedly be difficult to enact and requires a truly multi-disciplinary life-course approach involving systems spanning childhood and adolescence, and commencing at a time well before pregnancy is contemplated [54].

## Conclusions

While dietary intervention in pregnancy has been shown to improve maternal dietary behaviours, and have a modest effect on gestational weight gain, there is no evidence from this IPD MA that there is an effect on early childhood obesity or persistent effects on maternal weight after birth.

## Supplementary Information

**Additional file 1.** PRISMA IPD Checklist. Checklist of items to include when reporting an individual participant data meta-analysis.

**Additional file 2.** PICO Question and search strategy. Describing the participants, intervention, comparator and outcomes. Search terms for each search are presented.

## Data Availability

Additional IPDMA-related documents and requests for de-identified data (aggregate or individual participant level) may be requested by written application to the corresponding author and will be considered on an individual basis by the IPDMA author group.

## Abbreviations

aRR: Adjusted relative risk
BMI: Body mass index
CI: Confidence interval
DOHaD: Developmental origins of health and disease
FCS: Fully conditional specification
GA: Gestational age
GWG: Gestational weight gain
HC: Head circumference
IPD: Individual participant data
IPDMA: Individual participant data meta-analysis
iWIP: International Weight Management in Pregnancy Collaborative Group
MVN: Multivariate normal
PRISMA: Preferred Reporting Items for Systematic Reviews and Meta-Analyses
PROSPERO: International Prospective Register of Ongoing Systematic Reviews
RCT: Randomised controlled trial
REML: Restricted maximum likelihood
RR: Relative risk
SFTM: Skinfold thickness measurement
