# Supplementary material for: The effects of dietary and lifestyle interventions among pregnant women with overweight or obesity on early childhood outcomes: an individual participant data meta-analysis from randomised trials
Source: BMC Med. 2021 Jun 2;19:128. doi: 10.1186/s12916-021-01995-6 (PMC8170974; doi:10.1186/s12916-021-01995-6)
Supplement: Supplementary file 2 — Additional file 2. PICO Question and search strategy. Describing the participants, intervention, comparator and outcomes. Search terms for each search are presented. [file 12916_2021_1995_MOESM2_ESM.pdf]

PICO Question:

Effects of antenatal dietary and lifestyle interventions for women who are overweight and obese on longer term health outcomes for women and children at 3-5 years of age

P – Pregnant women who are overweight and obese

I – antenatal dietary and lifestyle/physical activity/exercise

C – standard care/control

O – maternal longer term health 3-5 years postnatal OR child/infant/preschool health/obesity

1) maternal metabolic syndrome diagnosis
2) childhood BMI >90th percentile for age

Search for Pubmed:

Pregnant women [MH] OR Pregnancy [MH] OR Pregnan* [TW] OR Maternal obesity [TW] OR Female [MH] AND Overweight [MH] AND Humans [MH]	Diet therapy [MH] OR overweight/diet therapy [MH] OR Diet* [TW] exercise [MH] OR exercise [TW]	Child, preschool [MH] AND Pediatric obesity [MH]	Randomized controlled trial [MH] OR Cohort studies [MH]

Pregnant women [MH] OR Pregnancy [MH] OR Pregnan* [TW] OR Maternal obesity [TW] OR Female [MH] AND Overweight [MH] AND Humans [MH]) AND (Diet therapy [MH] OR overweight/diet therapy [MH] OR Diet* [TW] OR exercise [MH] OR exercise [TW]) AND prenatal care [MH]

Search for PubMed Central:
Pregnant women [MH] OR Pregnancy [MH] OR Pregnan* [ALL] OR Maternal obesity [ALL] OR Female [MH] AND Overweight [MH] AND Humans [MH]) AND (Diet therapy [MH] OR overweight/diet therapy [MH] OR Diet* [ALL] OR exercise [MH] OR exercise [ALL]) AND prenatal care [MH]

(Pregnant women [MH] OR Pregnancy [MH] OR Pregnan* [ALL] OR Maternal obesity [ALL] OR Female [MH] AND Overweight [MH] AND Humans [MH]) AND (Diet therapy [MH] OR overweight/diet therapy [MH] OR Diet* [ALL] exercise [MH] OR exercise [ALL] OR Behaviour/Physiology [ALL] OR Birth weight/Physiology [ALL] OR Health Education* [ALL] OR Primary Prevention*[ALL] OR Life style* [ALL]) AND Cohort studies [MH]

Search for Embase:

(('pregnant woman'/exp OR 'pregnant woman' OR 'pregnant women' OR 'pregnancy'/exp OR 'child bearing' OR 'childbearing' OR 'gestation' OR 'gravidity' OR 'intrauterine pregnancy' OR 'labor presentation' OR 'labour presentation' OR 'pregnancy' OR 'pregnancy maintenance' OR 'pregnancy trimesters' OR 'maternal'/exp) AND ('obesity'/exp OR 'adipose tissue hyperplasia' OR 'adipositas' OR 'adiposity' OR 'alimentary obesity' OR 'body weight, excess' OR 'corpulency' OR 'fat overload syndrome' OR 'nutritional obesity' OR 'obesitas' OR 'obesity' OR 'overweight') OR 'maternal obesity'/exp OR 'maternal obesity' OR 'obesity, maternal') AND (('diet therapy'/exp OR 'diet intervention' OR 'diet therapy' OR 'diet treatment' OR 'dietary intervention' OR 'dietary therapy' OR 'dietary treatment' OR 'nutrition therapy' OR 'nutritional therapy') AND ('lifestyle modification'/exp OR 'life style change' OR 'life style changes' OR 'life style modification' OR 'life style modifications' OR 'lifestyle change' OR 'lifestyle changes' OR 'lifestyle modification' OR 'lifestyle modifications') OR 'physical activity'/exp OR 'activity, physical' OR 'physical activity' OR 'exercise'/exp OR 'biometric exercise' OR 'effort' OR 'exercise' OR 'exercise capacity' OR 'exercise performance' OR 'exercise training' OR 'exertion' OR 'fitness training' OR 'physical conditioning, human' OR 'physical effort' OR 'physical exercise' OR 'physical exertion') AND ('prenatal care'/exp OR 'ante natal care' OR 'antenatal care' OR 'antenatal control' OR 'prenatal care') AND ('standard care' OR 'control'/exp OR 'control' OR 'internal-external control' OR 'no treatment')

Search for Ovid medline:

(exp Pregnant women / or exp Pregnancy / or Pregnan* .af. or Maternal obesity .af. or exp Female / and exp Overweight / and exp Humans /) and (exp Diet therapy / or exp overweight/diet therapy or exp Diet* [ALL] exercise / or exercise .af. or Behaviour/Physiology .af. or Birth weight/Physiology .af. or Health Education* .af. or Primary Prevention*.af. or Life style* .af.) and exp Cohort studies /
